# Impact of Exercise on Tramadol-Conditioned Place Preference

**DOI:** 10.3390/brainsci15010089

**Published:** 2025-01-18

**Authors:** Haneen Amawi, Alaa M. Hammad, Aseel Abdullah Ibrahim, Nosyba Alsbih, Frank Scott Hall, Fawaz Alasmari, Bahaa Al-Trad

**Affiliations:** 1 Department of Clinical Pharmacy and Pharmacy Practice, College of Pharmacy, Yarmouk University, Irbid 21163, Jordan; 2 Department of Pharmacy, College of Pharmacy, Al-Zaytoonah University of Jordan, Amman 11733, Jordan; 3 Department of Biological Sciences, Faculty of Science, Yarmouk University, Irbid 21163, Jordanbahaa.tr@yu.edu.jo (B.A.-T.); 4 Department of Pharmacology and Experimental Therapeutics, College of Pharmacy and Pharmaceutical Sciences, University of Toledo, Toledo, OH 43614, USA; frank.hall@utoledo.edu; 5 Department of Pharmacology and Toxicology, College of Pharmacy, King Saud University, Riyadh 11451, Saudi Arabia; ffalasmari@ksu.edu.sa

**Keywords:** *Bdnf*, conditioned place preference, exercise, *Il-1β*, tramadol

## Abstract

Background: Tramadol (TRA) is an opioid that is used to manage moderate to severe pain. Long-term use of TRA can lead to the development of opioid use disorder. Objectives: This study investigates the role of forced exercise in reducing TRA-seeking behavior. Methods: Adult male rats (240–260 g) were divided into five groups; the control group received vehicle injections, the TRA group received TRA (75 mg/kg, i.p) every other day for 8 days, and three TRA–exercise groups were forced to run on a treadmill (60 min/day, 5 days/week) for 2, 4, or 6 weeks prior to conditioning with TRA. A tramadol-conditioned place preference (CPP) procedure assessed TRA reinforcement, after which all rats were euthanized, tissue extracted, and mRNA expression for brain-derived neurotrophic factor (*Bdnf*) and interleukin 1 beta (*Il-1β*) determined in hippocampus (Hipp), prefrontal cortex (PFC), and nucleus accumbens (NAc). Results: TRA-seeking behavior was seen in the TRA group and the 6 weeks forced exercise group. By contrast, forced exercise for 2 or 4 weeks attenuated TRA-seeking behavior. This attenuation was associated with a significant increase in *Bdnf* mRNA expression in the Hipp and NAc, but not the PFC. Additionally, the TRA-induced elevations in *Il-1β* mRNA expression were reversed by all durations of exercise in Hipp. However, only 2 and 4 weeks, but not 6 weeks, of exercise reduced elevations in PFC and NAc *Il-1β* expression. Conclusion: Forced exercise for 2 and 4 weeks attenuates TRA-seeking behavior partially through the regulation of *Bdnf* and *Il-1β* mRNA expression.

## 1. Introduction

Approximately 35.6 million of the world’s population between the ages of 15 and 64 suffer from substance use disorders. Opioids continue to cause the highest ratio of harm [1]. These medications are effective in reducing pain resulting from various illnesses and other medical conditions. However, their long-term misuse results in progression to substance use disorders in up to 50% of users [2]. Chronic exposure to these drugs leads to adaptive changes in the central nervous system, resulting in tolerance, physical dependence, sensitization, craving, and relapse [3]. Opioids do not directly affect dopamine systems; all drugs of abuse, directly or indirectly, activate the dopamine system, including opioids [4]. Drug dependence is a complex psychological behavior resulting from the interaction of several neurotransmitters, including dopamine and glutamate, in various brain regions [5,6]. Importantly, there is substantial evidence that the nucleus accumbens (NAc) is where dopamine-independent reinforcement occurs, suggesting that these brain regions receive multiple inputs that activate critical drug reinforcement circuits [7].

Another molecular mechanism that critically underlies drug dependence is the release of brain-derived neurotrophic factor (BDNF). BDNF is a neurotrophic factor that promotes neuronal survival, as well as the growth and differentiation of new neurons and synapses [8]. It is one of the few markers that was positively associated with the incubation of drug seeking over the course of drug abstinence, although most of these studies have focused on animal models of cocaine relapse [9,10]. Results from these studies show that during early abstinence, when levels of drug seeking are low, markers of BDNF, including the activity of its intracellular pathways (e.g., extracellular signal-regulated kinases (ERKs)), are decreased in several brain regions including the prefrontal cortex (PFC) and NAc [10,11]. In addition to these mechanisms, but perhaps interacting with them, neuroinflammation has a causal role in the pathological neural activity induced by drugs of abuse [12]. The overall effect of neuroinflammation is mediated, at least in part, by an increase in the production of pro-inflammatory cytokines, such as IL-1β, and TNF-α. Therefore, therapeutic strategies targeting the regulation or availability of inflammatory mediators might be used to prevent drug-seeking behavior during voluntary abstinence.

Tramadol (TRA), a centrally acting analgesic, is commonly prescribed to relieve moderate to severe pain. TRA has a dual mechanism of action: agonizing opioid receptors and blocking the reuptake of norepinephrine and serotonin in the central nervous system [13,14]. A previous assessment by the World Health Organization and the United States Drug Enforcement Agency concluded that TRA has a low potential for abuse [15], although this does not accord with all assessments [16]. A study of use patterns among Jordanian university students showed a pattern of self-medication and misuse of TRA that is likely to lead to substance use disorders [17]. TRA induces a craving response similar to that of oxycodone among opioid abusers [18]. As a result, many individuals who have become addicted to stronger opioid medications also misuse TRA. The vast majority of cases of TRA abuse (97%) occur in individuals with a history of abuse of other drugs [19]. Reports continue to confirm the potential of TRA misuse, as shown by an increase in the proportion of non-physician TRA use by patients of 56% between 2012 and 2015 [20].

Conditioned place preference (CPP) is used to assess the reinforcing potential of drugs [21]. It is used to investigate the neural mechanisms underlying drug dependence and drug-seeking behavior [22]. CPP occurs when a subject chooses a place associated with the administration of a drug, which is taken as indication of its rewarding and reinforcing effects [23]. The CPP model is widely used to examine the reinforcing effects of drugs, which indicate their potential for abuse and addiction [24]. Drugs that produce CPP are highly reinforcing, with abuse potential.

Although many tools for the prevention and treatment of drug-seeking behavior have been developed over the years, both pharmacologic and behavioral, these remain inadequate to solve the problem. Research continues to look for more effective options. Physical exercise has been shown to reduce cocaine-seeking behavior in the CPP paradigm [25]. Similar effects have been shown for the acquisition and extinction of methamphetamine CPP [26,27]. Thus, increased physical activity may be beneficial in the prevention and treatment of substance use disorders. In support of this hypothesis, it has been found that promoting physical activity during adolescence may protect both males and females from abusing cocaine [25]. Less work has been carried out to examine the effects of increased physical activity or exercise on opioid reinforcement. Thus, the main goal of the present work was to investigate, for the first time, the effects of exercise on TRA-seeking behavior in rats by assessing drug reinforcement using CPP. The effects of different durations of exercise on TRA-seeking behavior were also explored, as well as changes in the mRNA expression levels of *Bdnf* and *Il1b*, and in NAc and PFC as well as hippocampus (Hipp) to assess the potential mechanisms by which exercise might reduce TRA reinforcement.

## 2. Materials and Methods

**Figure 1:**
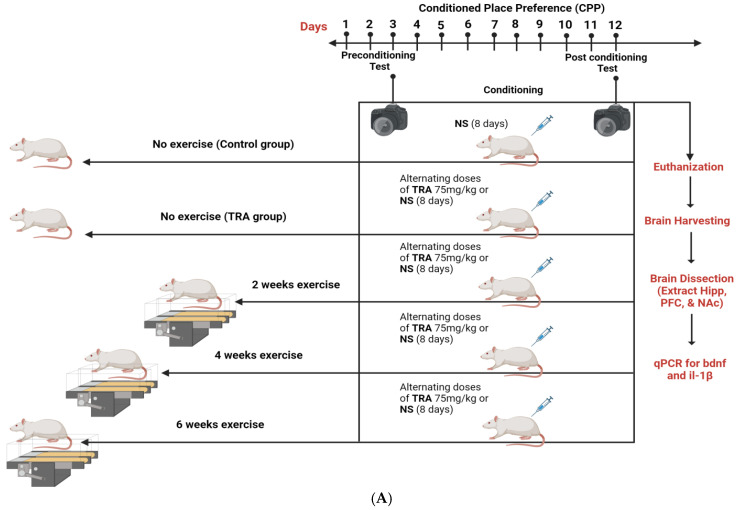

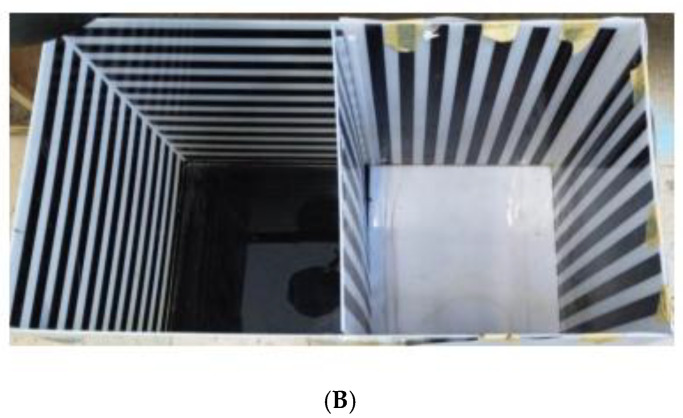
(**A**) The experimental design and timeline for the study. TRA; tramadol, NS; normal saline, Hipp; hippocampus, PFC; prefrontal cortex, NAc; nucleus accumbens, Il-1β; Interleukin-1-beta, bdnf; brain-derived neurotrophic factor and qPCR; quantitative PCR (this figure was generated using BioRender^®^ 2024). (**B**) Conditioning place preference apparatus.

All experimental procedures were approved by the Institutional Animal Care and Use Committee at Yarmouk University (IACUC/2022/8), dated 4 September 2022. Male Sprague Dawley (SD) rats (240–260 g) were obtained from the animal house of Yarmouk University. Animals were housed under standard laboratory conditions: temperature of 21–25 °C, 55% humidity, and a 12 h light–dark cycle. Each rat was housed alone in a 45 cm × 30 cm × 20 cm cage with wood shaving bedding and no environmental enrichment. Rats had free access to water and a standard chow diet. Rats were divided into five groups with 10 rats in each group: the control group received normal saline and underwent the CPP paradigm; the TRA–saline group received alternating doses of TRA (75 mg/kg i.p.) and saline injections for 8 days during the CPP paradigm; and three exercise groups were forced to run on a treadmill for 2, 4, or 6 weeks (60 min/day, 5 days/week) before undergoing CPP testing, and then each group received alternating doses of TRA (75 mg/kg i.p.) and saline injections for 8 days during CPP testing, as reported in previous studies [28,29]. TRA (Hikma Pharmaceuticals, Amman) was dissolved in saline and was given intraperitoneally (75 mg/kg i.p.). This was an effective dose for CPP based on another study [30]. The experimental design is shown in Figure 1A and the conditioning place preference apparatus in Figure 1B.

### 2.1. Chronic Daily Treadmill Exercise Regimen

A treadmill instrument was used to perform forced exercise on experimental rats. The treadmill is divided into five running lanes by plexiglass dividers. The treadmill was placed in a separate room from the housing room. All exercise subjects (n = 30 males, 10 in each exercise group) were adapted according to the same exercise model. The exercise was performed at the same time in the morning between 7:00 am and 10:00 am.

The treadmill was started for a duration of 10 min/day. The speed (15 m/min) was set within the range that has been utilized in previous studies on rats [31]. The rate was kept constant, and the duration of exercise was prolonged by 10 min/day until 60 min/day was reached. The rats were given a ten-minute break after the first half-hour of exercise. Rats in the three exercise groups were maintained on this daily exercise regime, 5 days per week, for 2, 4, and 6 weeks prior to the CPP test.

### 2.2. Conditioning Place Preference (CPP) Apparatus

The apparatus was made of rectangular plexiglass divided into two compartments (white and black) separated by a removable guillotine door, with standard dimensions for each compartment of 35 cm (width) × 35 cm (length) × 50 cm (height). The rats were placed in a colorless chamber at the beginning of the experiment, from which they could transfer between the two chambers. The white compartment had black vertical stripes with a rough white floor, while the black chamber had white horizontal stripes and a smooth black floor. A table lamp (18 w) was situated about 74 cm above the black compartment alongside a video camera placed above the apparatus to record the movement of the rats within the two compartments during the test. The time spent in each chamber was determined by an observer blinded to the experimental conditions, defining the location of the head as the location of the rat in the apparatus as described previously [32].

#### 2.2.1. Pre-Conditioning Phase

In this phase, the rats were allowed to roam freely in the CPP apparatus for 20 min/day on the first two days with the guillotine door open (the habituation phase). No treatment was given at this stage. To ensure an unbiased design, on Day 3, a digital camera was situated above the apparatus and a recording was made. The time spent in each chamber determined by an observer blinded to the experimental conditions, defining the location of the head as the location of the rat in the apparatus as described previously [32]. When rats displayed strong initial preference for any chamber for more than 67% of the total time, they were removed from the study [33].

#### 2.2.2. Conditioning Phase

Rats in the control group were injected with saline each day (1 mL/kg) for 8 days prior to placement in each chamber. In the four groups that received TRA, rats received injections of TRA (75 mg/kg i.p.) or saline (1 mL/kg; equivolume to the TRA injections) on alternate days (8 days, 4 doses of each) prior to placement in the chambers, one being the TRA+ chamber and one being the TRA- chamber. The exercise groups were treated in the same manner. TRA injections were given after the treadmill exercise session. On days four, six, eight, and ten, rats were treated with TRA and then confined to the TRA+ compartment for 30 min. On days five, seven, nine, and eleven, rats were treated with saline and then confined to the TRA- compartment for 30 min. Compartment location (right side or left side) paired with TRA was pseudorandomized, as in a previous study [34].

#### 2.2.3. Post-Conditioning Test

After the conditioning phase, a post-conditioning test was conducted on day twelve, in a manner identical to the pre-conditioning test. No exercise session was conducted on this day. During the test, the guillotine door between the two chambers was removed and rats were allowed to freely move in the two chambers for 20 min. This was recorded by a video camera. The time spent on each side was calculated in the same manner as the pre-conditioning test described above. The percentage of time spent in the drug-paired chamber was calculated through this equation: (time spent in the drug-paired chamber/the total time of the test) × 100.

## 3. Harvesting of Brain Tissue

Twenty-four hours after the post-conditioning test, rats were euthanized with diethyl ether and promptly decapitated using a guillotine. The brains were harvested immediately and stored at −80 °C for further analysis. A cryostat apparatus (CM1850, Leica, Wetzlar, Germany) was kept at −20 °C to keep the tissue frozen and was used for the dissection. The NAc (core and shell) (relative to bregma: +1.2–+2.2 mm) with a weight of 400–500 µg, the medial PFC (mPFC) (relative to bregma: +2.7–+3.7 mm) with a weight of 600–700 µg, and the Hipp (relative to bregma: −3.14–−4.16 mm) with a weight of 600–700 µg were obtained [35].

### 3.1. Gene Expression Analysis

Total RNA was isolated from the NAc, the Hipp, and the PFC using Monarch^®^ RNA Purification Kits (New England Biolabs, Ipswich, MA, USA). cDNA was then prepared using a commercial kit, SOLIcript^®^ RT (Tartu, Estonia) according to the manufacturer’s instructions, with 4 µg of total RNA from each sample used. A Nanodrop Quawell DNA/Protein Analyzer was used to quantify RNA and cDNA concentrations (Sunnyvale, CA, USA). A quantitative real-time polymerase chain reaction (qRT-PCR) was then performed with a BlasTaq 2× qPCR MasterMix kit (New England Biolabs, Ipswich, MA, USA). The reaction included 10 μL of 2× BlasTaqTM qPCR MasterMix, 0.5 μL each of forward and reverse primers, 2 μL of cDNA template, specific primers for the gene of interest, and nuclease-free H_2_O added to a final volume of 20 μL. The Glyceraldehyde 3-phosphate dehydrogenase (*Gapdh*) gene was used as the reference gene for normalization. The relative quantification of the expression of target mRNA in experimental groups with those of control subjects was carried out by the 2^−ΔΔCT^ method [36]. The primers were as follows: *Gapdh* forward ACGGGAAAC CCA TCA CCA T and reverse CCA GCA TCA CCC CAT TTGA [37], *Bdnf* forward AAGTCTGCATTACATTCCTCGA and reverse GTTTTCTGAAAGAGGGACAGTTTAT [38], and *Il-1β* forward ACC CAA GCA CCT TCT TTT CCT T and reverse TGC AGC TGT CTA ATG GGA ACA T [37].

### 3.2. Statistical Analysis

Percentage of time spent in the drug chamber as well as the qRT-PCR data for *Bdnf* and *Il-1β* were analyzed using one-way ANOVA followed by Tukey’s multiple means comparisons. All data were analyzed using GraphPad Prism 9.0; significance was set at *p* < 0.05.

## 4. Results

**Figure 2:**
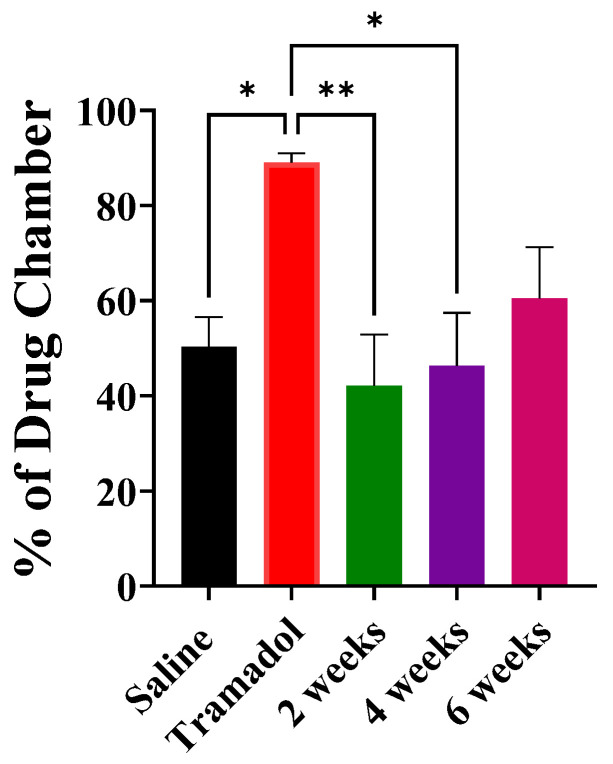
Percentage of time in the drug-paired chamber; after saline, tramadol (75 mg/kg × 4 days), and tramadol following 2, 4, or 6 weeks of forced exercise. Data are expressed as mean ± S.E.M. n = 10, * *p* < 0.05, ** *p* < 0.01.

Compared to the saline control group, four doses of tramadol treatment (75 mg/kg, i.p.) significantly increased the percentage of time spent in the drug-paired chamber (Figure 2). Exercise for 2 and 4 weeks significantly decreased the percentage of time spent in the drug-paired chamber, while exercise for 6 weeks did not significantly reduce the percentage of time spent in the drug-paired chamber. These group differences were confirmed by post hoc means comparisons following demonstration of a significant overall effect of the group in a one-way ANOVA [F (4, 45) = 4.471, *p* = 0.0040, Figure 2].

**Figure 3:**
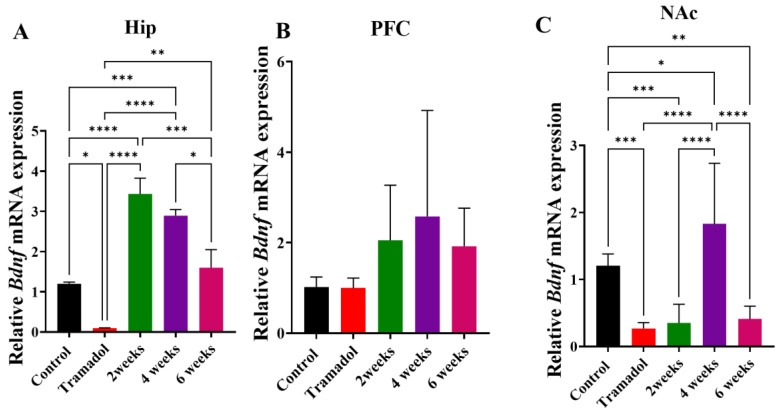
Relative mRNA expression of *Bdnf* in (**A**) hippocampus (Hip), (**B**) prefrontal cortex (PFC), and (**C**) nucleus accumbens (NAc) after tramadol treatment alone or in combination with 2, 4, or 6 weeks of physical exercise. Data are expressed as mean ± SEM (n = 5); * *p* < 0.05, ** *p* < 0.01, *** *p* < 0.001, **** *p* < 0.0001.

Figure 3 illustrates the effect of four doses of TRA (75 mg/kg for 4 days) with and without exercise on the mRNA expression of *Bdnf* in Hip (Figure 3A), PFC (Figure 3B), and NAc (Figure 3C). Tramadol significantly decreased the *Bdnf* mRNA expression, while exercise for 2 and 4 weeks upregulated the mRNA expression of *Bdnf*; indeed, these levels were above baseline (control) levels. However, exercise for 6 weeks did not significantly affect *Bdnf* mRNA expression. This effect was confirmed by one-way ANOVA, which indicated the significant main effect of treatment on relative bdnf mRNA expression [F (4, 20) = 25.09, *p* < 0.0001, Figure 3A], as well as post hoc means comparisons. No effect of tramadol or exercise was observed on mRNA expression of *Bdnf* in PFC, as shown in a one-way ANOVA [F (4, 20) = 1.53, *p* = 0.2308, Figure 3B]. Similar to the Hip results, a significant decrease was observed with tramadol and upregulation with 4 weeks of exercise but not with 2 or 6 weeks of exercise. This upregulation was not significantly different from baseline (control) values. This pattern of effects was confirmed by one-way ANOVA, which indicated a significant main effect of treatment on relative *Bdnf* mRNA expression [F (4, 20) = 10.56, *p* = 0.0001, Figure 3C], as well as post hoc means comparisons.

**Figure 4:**
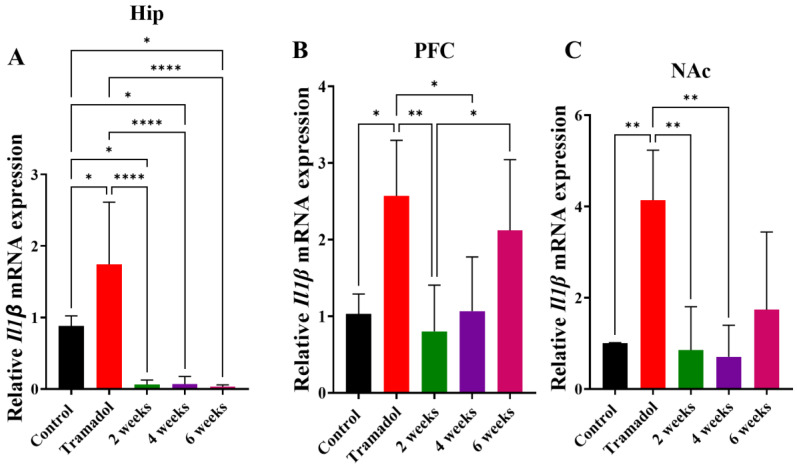
Relative mRNA expression of *Il-1β* in (**A**) hippocampus (Hip), (**B**) prefrontal cortex (PFC), and (**C**) nucleus accumbens (NAc) brain regions after tramadol treatment alone or in combination with 2, 4, or 6 weeks of physical exercise. Data are expressed as mean ± SEM (n = 5); * *p* < 0.05, ** *p* < 0.01 and **** *p* < 0.0001.

Figure 4 illustrates the effect of tramadol conditioning with or without exercise for either 2, 4, or 6 weeks on the mRNA expression of *Il-1β* in Hip (Figure 4A), PFC (Figure 4B), and NAc (Figure 4C). Tramadol significantly increased *Il-1β* mRNA expression in HIP, while exercise for 2, 4, and 6 weeks reduced mRNA expression of *Il-1β* below baseline (control) values. This effect was confirmed by one-way ANOVA, which indicated the significant main effect of treatment on relative *Il-1β* mRNA expression [F (4, 20) = 13.18, *p* < 0.0001, Figure 4A], followed by post hoc means comparisons. Similar data were obtained for the PFC, where tramadol upregulated *Il-1β* mRNA expression, and 2 and 4 weeks of exercise, but not 6 weeks, reduced the expression as indicated by one-way ANOVA [F (4, 20) = 6.58, *p* = 0.0015, Figure 4B]. A significant increase was also observed with tramadol in the NAC with restored expression after 2 and 4 weeks of exercise, but not with 6 weeks of exercise. This is shown by the significant effect of treatment in a one-way ANOVA for relative *Il-1β* mRNA expression [F (4, 20) = 8.23, *p* = 0.0006, Figure 4C].

## 5. Discussion

In this study, TRA (4 × 75 mg/kg i.p on alternate days) induced CPP, while forced exercise for 2 or 4 weeks, but not 6 weeks, reduced TRA-induced CPP, indicating a significant inhibition of drug-seeking behavior by exercise. Furthermore, TRA decreased relative mRNA expression of *Bdnf* in Hipp and NAc, but not PFC. This effect was reversed by exercise. TRA increased the relative mRNA expression of *Il-1β*, TRA in Hipp, NAc, and PFC, while exercise attenuated this effect. These data indicate that the reinforcing effects of TRA can be reduced by moderate exercise, but perhaps not more strenuous exercise, and that these effects may involve changes in *Bdnf* and *Il-1β*.

Previously, it was shown that TRA is associated with both abuse [39] and serious side effects [40,41], despite perceptions of its relative safety compared to other opioids. TRA can induce a preference for environments associated with its administration, which was confirmed by previous CPP studies in rodents suggestive of strong reinforcing effects and addictive potential [42]. Additionally, a high rate of non-therapeutic use (e.g., abuse) was also reported in humans [43]. In our results, TRA administered 4 times to rats at a dose of 75 mg/kg i.p. on alternate days resulted in significant drug-seeking behavior (e.g., CPP; seeking out the environment previously paired with the drug). This is consistent with previous reports. For example, a previous study used CPP to assess drug-seeking behavior induced by TRA. Different doses of TRA were examined (18.75, 37.5, and 75 mg/kg). The results indicated that TRA produced a statistically significant CPP, with the two highest doses inducing comparable effects to those induced by morphine (5 mg/kg). Thus, although a less potent drug, higher doses should be considered to have abuse potential. Moreover, the CPP results indicate that TRA has a greater potential for abuse than was originally thought [30]. Another study confirmed that TRA significantly increased the time spent in the drug-paired compartment post-conditioning compared to pre-conditioning [44].

Exercise is currently considered to be a beneficial adjunctive treatment for numerous chronic and complex diseases. Overall, exercise is considered to enhance well-being in general, as stated in the World Health Organization report 2020 [45], although many of those actions may have a more specific basis. Physical activity is recommended as a component of healthy lifestyle to protect against coronary heart disease (CHD) and cerebrovascular diseases [46] and can have significant health advantages for obese patients [47]. It is especially preferred as it is not associated with many side effects compared with pharmacological treatments [48]. Additionally, as a part of cognitive behavioral therapies, exercise can improve treatment outcomes for many depressed patients [48]. Participating in physical activities is a form of treatment because it helps to decrease mental health problems and improves social communication and self-esteem [49]. Moreover, exercise produces changes in metabolism and general inflammatory state [50], which may be quite helpful for conditions involving chronic inflammation. Thus, exercise can be beneficial for both physiological and psychological problems and would also be expected to have benefits for individuals in treatment for addiction given the role of inflammatory signaling in addiction.

Related to drug-seeking behavior, it was shown that physical activity in the form of aerobic exercise decreases drug self-administration and other measures of drug-seeking behavior. Studies show that exercise has positive effects in the treatment of drug abuse in humans [51,52]. This has been confirmed in animal models as well. For instance, running in an activity wheel or on a treadmill decreases the self-administration of nicotine [53], cocaine [54], methamphetamine, MDMA, and methylone [55]. Furthermore, exercise decreases the resumption of drug seeking after a period of abstinence. Moreover, aerobic exercise produces changes in central dopamine and opioid systems, which may explain such reductions in drug seeking and drug intake [56]. A previous clinical study of 25 participants in a 12-week exercise program showed a significant reduction in alcohol consumption in an exercise group compared to control subjects [57]. Changes in body mindfulness and an increase in energy level were observed.

Animal experiments have revealed promising treatment impacts of exercise on drug-seeking behaviors. For example, voluntary wheel running decreased reinstatement of cocaine-seeking behavior precipitated by exposure to cocaine and cocaine-paired cues [54,58]. Animal studies have shown that aerobic exercise reduces stimulus-driven drug seeking and decreases escalation and reinstatement of drug-seeking behavior [59]. It has been shown that mice that exercised on a treadmill and performed physical activity consumed alcohol significantly less than mice that did not exercise. In males, this effect was not as significant as in females, but it shows that mice change their pattern of alcohol intake when they increase physical activity [60].

Consistent with the above-mentioned reports, our results confirmed potential benefits of exercise in reversing the TRA-seeking behavior. Firstly, this work shows that TRA clearly produced drug-seeking behavior and has more abuse potential than widely thought. Secondly, two and four weeks of exercise significantly reduced TRA-seeking behavior in rats and, thus, is consistent with the potential of exercise as a non-pharmacological alternative or adjunctive treatment for drug use disorders, including opioid use disorders. It will be important to explore these actions for other opioids, including more potent opioid agonists, such as fentanyl. Physical activity can work as a treatment to improve brain diseases and further improve social aspects of life, an idea which can be extended to the treatment of addiction. By working on self-image in youngsters, exercise has a preventive effect on drug abuse [61], although the present data suggest that physiological effects beyond these psychological effects may contribute to these outcomes.

It must also be stated that these findings suggest that some exercise parameters appear to affect whether or not positive outcomes are seen. Our results showed that exercise for six weeks did not significantly decrease TRA-seeking behavior in rats. This longer duration of physical activity resulted in the disappearance of the benefits gained at 2 and 4 weeks of exercise. Since we did not explore changes in physiological parameters over the course of exercise, it is possible that the physiological consequences of the forced exercise may have changed at this longer time point. Thus, there may be a u-shaped response here where a certain duration of forced physical activity is beneficial, but beyond a certain point, this changes. In this regard, it should be noted that this was forced exercise; it will be important to compare the effects of voluntary exercise to those of forced exercise on these outcomes. Previously, it has been shown that the intensity of physical exercise or its forced or voluntary nature has different effects on drug taking. For example, a decrease in alcohol intake was observed in a strain of mice with a high preference for alcohol (C57BL/6J mice) after voluntary moderate physical activity. However, higher-speed forced physical activity increased alcohol intake in the same strain of mice [62]. Additionally, higher levels of endogenous opioids were detected following strenuous exercise, interpreted as a potential for “auto-addiction” [63]. Thus, while moderate exercise can have therapeutic benefits in inhibiting drug-seeking behavior, strenuous levels of exercise might increase seeking behavior by triggering the release of endorphins and dopamine, which can increase reinforcement, making individuals more likely to seek out rewarding experiences [64]. Although the intensity of exercise was not altered in the current experiment, the consequences of exercise may have changed at longer durations of forced exercise exposure in a similar manner, something that should be explored in the future. More research is needed to investigate the effects of short versus longer, moderate versus strenuous, and voluntary versus forced exercise on drug seeking and other drug-related phenotypes.

In the present results, TRA reduced *Bdnf* expression while exercise increased *Bdnf* expression, albeit in a brain region-specific manner. Two and four weeks of exercise, but not six weeks, significantly reversed TRA-induced decreases in bdnf and increased the relative mRNA levels of bdnf in the hippocampus. No significant changes were observed in the PFC, and only 4 weeks of exercise significantly increased relative mRNA levels of bdnf in NAc. Physical exercise has a lot of effects on the brain, as it improves neural connections in areas significant for learning and memory such as the hippocampus. Neurogenesis is stimulated by exercise as well [65]. A study on rats showed that exercise itself does not cause neurogenesis per se, but is responsible for recuperation after alcohol-induced damage [66], allowing a greater potential for neurogenesis. Exercise might do so by promoting growth factors, such as BDNF, which help maintain neural connections and act as a protective factor for neuronal survival [67]. However, different studies on exercise and addictive drugs found different results for effects on bdnf levels that were also brain region dependent. For example, previous research showed that cocaine exposure increases BDNF signaling in the NAc [68], and both aerobic [69] and resistance [70] exercise decreases bdnf gene expression in cocaine-exposed rats. Another study indicated that resistance exercise decreases cocaine self-administration and reduces bdnf mRNA expression in the NAc [71]. A decline in hippocampal, but not PFC, bdnf expression after morphine treatment in abstinent rats was observed [72]. Another study indicated that exercise increases BDNF in both humans and animal models [73]. It has been shown that exercise induced an upregulation of the BDNF in the hippocampus and might therefore play an important role in the enhancement of cognitive functions in humans by exercise [74]. The authors concluded that physical activity could increase the availability of bdnf to these cells by upregulating its expression in the hippocampus, and as a consequence, exercise might increase the brain’s resistance to damage [75]. Furthermore, it has been reported that as little as 6 h of voluntary wheel running results in a significant upregulation of the hippocampal bdnf mRNA expression in rats, which remained elevated after 12 h after voluntary running [76]. Thus, our results are consistant with the majority of previous studies indicating that exercise could induce an increase in bdnf levels in some brain regions, especially the hippocampus.

The connection between substance abuse and neuroinflammation has been confirmed previously in numerous studies. Neuroinflammation was found to induce neural adaptations following chronic exposure to drugs of abuse [12,77]. IL-1β, a pro-inflammatory cytokine, has been shown to be released in response to alcohol [78], cannabinoids [79], and opioids [80,81,82]. IL-1β may modulate the reinforcing effects of substances by influencing the release of dopamine, which is associated with drug reward and drug reinforcement. Our results showed that TRA induced significant elevations in *Il-1β* mRNA expression in the three tested brain regions (Hipp, PFC, and NAc). Exercising, especially for 2 and 4 weeks, reversed TRA-induced neuroinflammation as indicated by the reduction in the *Il-1β* mRNA levels in the three brain regions; indeed, these reductions were below control values. This is consistent with previous reports that showed the ability of physical activities to attenuate neuroinflammation in several chronic neurological disorders [83,84]. For example, 3 weeks of voluntary running exercise effectively normalized the elevated hippocampal levels of IL-1β and TNF-α in Tg2576 Alzheimer transgenic mice [85]. Traumatic brain injury-induced elevation in IL-1β and TNF-α was also reversed by 4 weeks of treadmill physical exercising in male Wistar rats [86].

This study has some limitations that should be considered. One limitation of our findings is the relatively small sample sizes in molecular investigations (n = 5 per group), which may have constrained our ability to detect smaller effects. However, the substantial effect sizes observed for many of our results, both for *bdnf* and *Il-1β* mRNA relative expression, support the reliability of the main observations presented. Furthermore, no measurement of intensity, assessment of aerobic capacity or lactate testing, or assessment of the relation to the anaerobic threshold was conducted; more studies are warranted to examine these effects. Although exercise can change locomotor capacity, CPP is not generally affected by such confounding effects since it measures the distribution of activity rather than being dependent on a measure of motor capacity (see discussion in [24]).

## 6. Conclusions

The current research focused on the role of different levels of exercise on reversing TRA-seeking behavior using a CPP rat model. TRA clearly produced reinforcing effects, suggestive of additive potential, while exercise reduced these effects. Specifically, results showed that 2 and 4 weeks, but not 6 weeks, of exercise reduced the time spent in the TRA-paired chamber, indicating significant inhibition of drug-seeking behavior. Further, molecular changes in bdnf and *Il-1β* mRNA expression levels in different brain regions (NAc, PFC, and Hipp) accompanied TRA conditioning, and these effects were reversed by exercise. The results suggest that moderate exercise might be beneficial for limiting TRA abuse potential. Further molecular studies are needed to understand the exact mechanisms by which exercising alters neurotransmission, neurogenesis, and neuro-inflammatory processes, as well as the optimal exercise conditions for producing such effects.

## Data Availability

All data generated or analyzed during this study are included in the published article.

## References

[1] Akerele E. (2022). Global Drug Use. Substance and Non-Substance Related Addictions: A Global Approach.

[2] Dydyk A.M., Jain N.K., Gupta M. (2023). Opioid use disorder. StatPearls [Internet].

[3] Justinova Z., Panlilio L.V., Goldberg S.R. (2009). Drug addiction. Behav. Neurobiol. Endocannabinoid Syst..

[4] Pierce R.C., Kumaresan V. (2006). The mesolimbic dopamine system: The final common pathway for the reinforcing effect of drugs of abuse?. Neurosci. Biobehav. Rev..

[5] Volkow N.D., Boyle M. (2018). Neuroscience of addiction: Relevance to prevention and treatment. Am. J. Psychiatry.

[6] Koob G.F., Volkow N.D. (2016). Neurobiology of addiction: A neurocircuitry analysis. Lancet Psychiatry.

[7] Nestler E.J. (2005). Is there a common molecular pathway for addiction?. Nat. Neurosci..

[8] Sanaeifar F., Pourranjbar S., Pourranjbar M., Ramezani S., Mehr S.R., Wadan A.-H.S., Khazeifard F. (2024). Beneficial effects of physical exercise on cognitive-behavioral impairments and brain-derived neurotrophic factor alteration in the limbic system induced by neurodegeneration. Exp. Gerontol..

[9] Geoffroy H., Noble F. (2017). BDNF during withdrawal. Vitam. Horm..

[10] Buzin N.R. (2014). Role of BDNF-TrkB Signaling in Cocaine Addiction. Ph.D. Thesis.

[11] Sun W.-L., Quizon P.M., Zhu J. (2016). Molecular mechanism: ERK signaling, drug addiction, and behavioral effects. Prog. Mol. Biol. Transl. Sci..

[12] Kohno M., Link J., Dennis L.E., McCready H., Huckans M., Hoffman W.F., Loftis J.M. (2019). Neuroinflammation in addiction: A review of neuroimaging studies and potential immunotherapies. Pharmacol. Biochem. Behav..

[13] Barakat A. (2019). Revisiting tramadol: A multi-modal agent for pain management. CNS Drugs.

[14] Baldo B.A. (2018). Opioid analgesic drugs and serotonin toxicity (syndrome): Mechanisms, animal models, and links to clinical effects. Arch. Toxicol..

[15] Vearrier D., Grundmann O. (2021). Clinical pharmacology, toxicity, and abuse potential of opioids. J. Clin. Pharmacol..

[16] Babalonis S., Lofwall M.R., Nuzzo P.A., Siegel A.J., Walsh S.L. (2013). Abuse liability and reinforcing efficacy of oral tramadol in humans. Drug Alcohol Depend..

[17] Malak M., AbuKamel A. (2019). Self-medication Practices among University Students in Jordan. Malays. J. Med. Health Sci..

[18] Edinoff A.N., Kaplan L.A., Khan S., Petersen M., Sauce E., Causey C.D., Cornett E.M., Imani F., Moghadam O.M., Kaye A.M. (2021). Full opioid agonists and tramadol: Pharmacological and clinical considerations. Anesthesiol. Pain Med..

[19] Jovanović-Čupić V., Martinović Ž., Nešić N. (2006). Seizures associated with intoxication and abuse of tramadol. Clin. Toxicol..

[20] Fleming M.L., Driver L., Sansgiry S.S., Abughosh S.M., Wanat M., Sawant R.V., Ferries E., Reeve K., Todd K.H. (2017). Physicians’ intention to prescribe hydrocodone combination products after rescheduling: A theory of reasoned action approach. Res. Soc. Adm. Pharm..

[21] Tzschentke T.M. (2007). Review on CPP: Measuring reward with the conditioned place preference (CPP) paradigm: Update of the last decade. Addict. Biol..

[22] Cami J., Farré M. (2003). Drug addiction. N. Engl. J. Med..

[23] Childs E., de Wit H. (2009). Amphetamine-induced place preference in humans. Biol. Psychiatry.

[24] Tzschentke T.M. (1998). Measuring reward with the conditioned place preference paradigm: A comprehensive review of drug effects, recent progress and new issues. Prog. Neurobiol..

[25] Thanos P.K., Tucci A., Stamos J., Robison L., Wang G.-J., Anderson B.J., Volkow N.D. (2010). Chronic forced exercise during adolescence decreases cocaine conditioned place preference in Lewis rats. Behav. Brain Res..

[26] Kitanaka N., Kitanaka J., Hall F.S., Uhl G.R., Watabe K., Kubo H., Takahashi H., Takemura M. (2012). Attenuation of methamphetamine-induced conditioned place preference in mice after a drug-free period and facilitation of this effect by exposure to a running wheel. J. Exp. Neurosci..

[27] Kitanaka N., Kitanaka J., Hall S., Okumura S., Sakamoto T., Uhl G., Takemura M. (2017). How the histamine N-methyltransferase inhibitor metoprine alleviates methamphetamine reward. J. Addict. Med. Ther. Sci..

[28] Hammad A.M., Amawi H., Hall F.S., Tiwari A.K., Al-Trad B. (2023). Effect of amoxicillin/clavulanic acid in attenuating pregabalin-induced condition place preference. Behav. Brain Res..

[29] Soares-Cardoso C., Leal S., Sá S.I., Dantas-Barros R., Dinis-Oliveira R.J., Faria J., Barbosa J. (2024). Unraveling the Hippocampal Molecular and Cellular Alterations behind Tramadol and Tapentadol Neurobehavioral Toxicity. Pharmaceuticals.

[30] Sprague J.E., Leifheit M., Selken J., Milks M.M., Kinder D.H., Nichols D.E. (2002). In vivo microdialysis and conditioned place preference studies in rats are consistent with abuse potential of tramadol. Synapse.

[31] Hosseini M., Alaei H.A., Naderi A., Sharifi M.R., Zahed R. (2009). Treadmill exercise reduces self-administration of morphine in male rats. Pathophysiology.

[32] Hammad A.M., Alasmari F., Sari Y. (2021). Effect of Modulation of the Astrocytic Glutamate Transporters’ Expression on Cocaine-Induced Reinstatement in Male P Rats Exposed to Ethanol. Alcohol Alcohol..

[33] Hammad A.M., Alasmari F., Althobaiti Y.S., Sari Y. (2017). Modulatory effects of Ampicillin/Sulbactam on glial glutamate transporters and metabotropic glutamate receptor 1 as well as reinstatement to cocaine-seeking behavior. Behav. Brain Res..

[34] Cunningham C.L., Gremel C.M., Groblewski P.A. (2006). Drug-induced conditioned place preference and aversion in mice. Nat. Protoc..

[35] Hammad A.M., Sari Y. (2020). Effects of Cocaine Exposure on Astrocytic Glutamate Transporters and Relapse-Like Ethanol-Drinking Behavior in Male Alcohol-Preferring Rats. Alcohol Alcohol.

[36] Livak K.J., Schmittgen T.D. (2001). Analysis of relative gene expression data using real-time quantitative PCR and the 2−ΔΔCT method. Methods.

[37] Apkarian A.V., Lavarello S., Randolf A., Berra H.H., Chialvo D.R., Besedovsky H.O., del Rey A. (2006). Expression of IL-1beta in supraspinal brain regions in rats with neuropathic pain. Neurosci. Lett..

[38] Jaehne E.J., Kent J.N., Antolasic E.J., Wright B.J., Spiers J.G., Creutzberg K.C., De Rosa F., Riva M.A., Sortwell C.E., Collier T.J. (2022). Behavioral phenotyping of a rat model of the BDNF Val66Met polymorphism reveals selective impairment of fear memory. Transl. Psychiatry.

[39] Tjäderborn M., Jönsson A.K., Ahlner J., Hägg S. (2009). Tramadol dependence: A survey of spontaneously reported cases in Sweden. Pharmacoepidemiol. Drug Saf..

[40] Tjäderborn M., Jönsson A.K., Hägg S., Ahlner J. (2007). Fatal unintentional intoxications with tramadol during 1995–2005. Forensic Sci. Int..

[41] Sansone R.A., Sansone L.A. (2009). Tramadol: Seizures, serotonin syndrome, and coadministered antidepressants. Psychiatry.

[42] Cha H.J., Song M.J., Lee K.W., Kim E.J., Kim Y.H., Lee Y., Seong W.K., Hong S.I., Jang C.G., Yoo H.S. (2014). Dependence potential of tramadol: Behavioral pharmacology in rodents. Biomol. Ther..

[43] Senay E.C., Adams E.H., Geller A., Inciardi J.A., Muñoz A., Schnoll S.H., Woody G.E., Cicero T.J. (2003). Physical dependence on Ultram® (tramadol hydrochloride): Both opioid-like and atypical withdrawal symptoms occur. Drug Alcohol Depend..

[44] Abdel-Ghany R., Nabil M., Abdel-Aal M., Barakat W. (2015). Nalbuphine could decrease the rewarding effect induced by tramadol in mice while enhancing its antinociceptive activity. Eur. J. Pharmacol..

[45] Morris T., Roychowdhury D. (2020). Physical activity for health and wellbeing: The role of motives for participation. Health Psychol. Rep..

[46] Carnethon M.R. (2009). Physical activity and cardiovascular disease: How much is enough?. Am. J. Lifestyle Med..

[47] McInnis K.J. (2003). Diet, exercise, and the challenge of combating obesity in primary care. J. Cardiovasc. Nurs..

[48] Takahashi H., Kawaguchi M., Kitamura K., Narumiya S., Kawamura M., Tengan I., Nishimoto S., Hanamure Y., Majima Y., Tsubura S. (2018). An exploratory study on the anti-inflammatory effects of fucoidan in relation to quality of life in advanced cancer patients. Integr. Cancer Ther..

[49] Lubans D., Richards J., Hillman C., Faulkner G., Beauchamp M., Nilsson M., Kelly P., Smith J., Raine L., Biddle S. (2016). Physical activity for cognitive and mental health in youth: A systematic review of mechanisms. Pediatrics.

[50] Beavers K.M., Brinkley T.E., Nicklas B.J. (2010). Effect of exercise training on chronic inflammation. Clin. Chim. Acta.

[51] Smith M.A., Lynch W.J. (2012). Exercise as a potential treatment for drug abuse: Evidence from preclinical studies. Front. Psychiatry.

[52] Zhang L., Yuan T.-F. (2019). Exercise and substance abuse. Int. Rev. Neurobiol..

[53] Sanchez V., Moore C.F., Brunzell D.H., Lynch W.J. (2013). Effect of wheel-running during abstinence on subsequent nicotine-seeking in rats. Psychopharmacology.

[54] Zlebnik N.E., Saykao A.T., Carroll M.E. (2014). Effects of combined exercise and progesterone treatments on cocaine seeking in male and female rats. Psychopharmacology.

[55] Aarde S.M., Miller M.L., Creehan K.M., Vandewater S.A., Taffe M.A. (2015). One day access to a running wheel reduces self-administration of D-methamphetamine, MDMA and methylone. Drug Alcohol Depend..

[56] Lynch W.J., Peterson A.B., Sanchez V., Abel J., Smith M.A. (2013). Exercise as a novel treatment for drug addiction: A neurobiological and stage-dependent hypothesis. Neurosci. Biobehav. Rev..

[57] Brown R.A., Prince M.A., Minami H., Abrantes A.M. (2016). An exploratory analysis of changes in mood, anxiety and craving from pre-to post-single sessions of exercise, over 12 weeks, among patients with alcohol dependence. Ment. Health Phys. Act..

[58] Smith M.A., Witte M.A. (2012). The effects of exercise on cocaine self-administration, food-maintained responding, and locomotor activity in female rats: Importance of the temporal relationship between physical activity and initial drug exposure. Exp. Clin. Psychopharmacol..

[59] Zlebnik N.E., Carroll M.E. (2015). Prevention of the incubation of cocaine seeking by aerobic exercise in female rats. Psychopharmacology.

[60] Ehringer M.A., Hoft N.R., Zunhammer M. (2009). Reduced alcohol consumption in mice with access to a running wheel. Alcohol.

[61] Collingwood T.R., Sunderlin J., Reynolds R., Kohl III H.W. (2000). Physical training as a substance abuse prevention intervention for youth. J. Drug Educ..

[62] Pichard C., Gorwood P.A., Hamon M., Cohen-Salmon C. (2009). Differential effects of free versus imposed motor activity on alcohol consumption in C57BL/6J versus DBA/2J mice. Alcohol.

[63] Adams J., Kirkby R.J. (2002). Excessive exercise as an addiction: A review. Addict. Res. Theory.

[64] Dishman R.K., Berthoud H.R., Booth F.W., Cotman C.W., Edgerton V.R., Fleshner M.R., Gandevia S.C., Gomez-Pinilla F., Greenwood B.N., Hillman C.H. (2006). Neurobiology of exercise. Obesity.

[65] Bardo M.T., Compton W.M. (2015). Does physical activity protect against drug abuse vulnerability?. Drug Alcohol Depend..

[66] Stevenson J.R. (2008). An Assessment of the Neurobiological and Behavioral Changes That Occur During Abstinence Following Chronic Alcohol Drinking. Ph.D. Thesis.

[67] Maynard M.E., Leasure J.L. (2013). Exercise enhances hippocampal recovery following binge ethanol exposure. PLoS ONE.

[68] Lynch W.J., Bakhti-Suroosh A., Abel J.M., Davis C. (2021). Shifts in the neurobiological mechanisms motivating cocaine use with the development of an addiction-like phenotype in male rats. Psychopharmacology.

[69] Peterson A.B., Hivick D.P., Lynch W.J. (2014). Dose-dependent effectiveness of wheel running to attenuate cocaine-seeking: Impact of sex and estrous cycle in rats. Psychopharmacology.

[70] Strickland J.C., Abel J.M., Lacy R.T., Beckmann J.S., Witte M.A., Lynch W.J., Smith M.A. (2016). The effects of resistance exercise on cocaine self-administration, muscle hypertrophy, and BDNF expression in the nucleus accumbens. Drug Alcohol Depend..

[71] Smith M.A., Fronk G.E., Abel J.M., Lacy R.T., Bills S.E., Lynch W.J. (2018). Resistance exercise decreases heroin self-administration and alters gene expression in the nucleus accumbens of heroin-exposed rats. Psychopharmacology.

[72] Shahroodi A., Mohammadi F., Vafaei A.A., Miladi-Gorji H., Bandegi A.R., Rashidy-Pour A. (2020). Impact of different intensities of forced exercise on deficits of spatial and aversive memory, anxiety-like behavior, and hippocampal BDNF during morphine abstinence period in male rats. Metab. Brain Dis..

[73] Pilc J. (2010). The effect of physical activity on the brain derived neurotrophic factor: From animal to human studies. J. Physiol. Pharmacol..

[74] Chen M.J., Russo-Neustadt A.A. (2009). Running exercise-induced up-regulation of hippocampal brain-derived neurotrophic factor is CREB-dependent. Hippocampus.

[75] Neeper S.A., Gómez-Pinilla F., Choi J., Cotman C.W. (1996). Physical activity increases mRNA for brain-derived neurotrophic factor and nerve growth factor in rat brain. Brain Res..

[76] Groves-Chapman J.L., Murray P.S., Stevens K.L., Monroe D.C., Koch L.G., Britton S.L., Holmes P.V., Dishman R.K. (2011). Changes in mRNA levels for brain-derived neurotrophic factor after wheel running in rats selectively bred for high-and low-aerobic capacity. Brain Res..

[77] Clark K.H., Wiley C.A., Bradberry C.W. (2013). Psychostimulant abuse and neuroinflammation: Emerging evidence of their interconnection. Neurotox. Res..

[78] Lippai D., Bala S., Csak T., Kurt-Jones E.A., Szabo G. (2013). Chronic alcohol-induced microRNA-155 contributes to neuroinflammation in a TLR4-dependent manner in mice. PLoS ONE.

[79] Moretti S., Castelli M., Franchi S., Raggi M.A., Mercolini L., Protti M., Somaini L., Panerai A.E., Sacerdote P. (2014). Δ^9^-Tetrahydrocannabinol-induced anti-inflammatory responses in adolescent mice switch to proinflammatory in adulthood. J. Leukoc. Biol..

[80] Liu L. (2011). CNS Immune Signalling and Drug Addiction: The Role of Interleukin-1 Beta. Ph.D. Thesis.

[81] Achur R.N., Freeman W.M., Vrana K.E. (2010). Circulating cytokines as biomarkers of alcohol abuse and alcoholism. J. Neuroimmune Pharmacol..

[82] Campbell L.A., Avdoshina V., Rozzi S., Mocchetti I. (2013). CCL5 and cytokine expression in the rat brain: Differential modulation by chronic morphine and morphine withdrawal. Brain Behav. Immun..

[83] Spielman L.J., Little J.P., Klegeris A. (2016). Physical activity and exercise attenuate neuroinflammation in neurological diseases. Brain Res. Bull..

[84] Svensson M., Lexell J., Deierborg T. (2015). Effects of physical exercise on neuroinflammation, neuroplasticity, neurodegeneration, and behavior: What we can learn from animal models in clinical settings. Neurorehabilit. Neural Repair.

[85] Nichol K.E., Poon W.W., Parachikova A.I., Cribbs D.H., Glabe C.G., Cotman C.W. (2008). Exercise alters the immune profile in Tg2576 Alzheimer mice toward a response coincident with improved cognitive performance and decreased amyloid. J. Neuroinflam..

[86] Mota B.C., Pereira L., Souza M.A., Silva L.F., Magni D.V., Ferreira A.P., Oliveira M.S., Furian A.F., Mazzardo-Martins L., Silva M.D. (2012). Exercise pre-conditioning reduces brain inflammation and protects against toxicity induced by traumatic brain injury: Behavioral and neurochemical approach. Neurotox. Res..

